# Treatment and Prognosis of Patients with Lung Cancer and Combined Interstitial Lung Disease

**DOI:** 10.3390/cancers15153876

**Published:** 2023-07-30

**Authors:** Charlotte Mauclet, Michaël V. Dupont, Kerwin Roelandt, Maxime Regnier, Monique Delos, Lionel Pirard, Thierry Vander Borght, Caroline Dahlqvist, Antoine Froidure, Benoît Rondelet, Jean Vanderick, Vincent Remouchamps, Fabrice Duplaquet, Sebahat Ocak

**Affiliations:** 1 Division of Pulmonology, Clinique Saint-Luc Bouge, Rue Saint-Luc, 8, 5004 Namur, Belgium; 2 Division of Radiology, CHU UCL Namur (Godinne Site), Université Catholique de Louvain (UCLouvain), Avenue G. Thérasse, 1, 5530 Yvoir, Belgium; michael.dupont@chuuclnamur.uclouvain.be (M.V.D.); kerwin.roelandt@chrhautesenne.be (K.R.); 3 Scientific Support Unit, CHU UCL Namur (Godinne Site), Université Catholique de Louvain (UCLouvain), Avenue G. Thérasse, 1, 5530 Yvoir, Belgium; maxime.regnier@unamur.be; 4 Division of Pathology, CHU UCL Namur (Godinne Site), Université Catholique de Louvain (UCLouvain), Avenue G. Thérasse, 1, 5530 Yvoir, Belgium; monique.delos@chuuclnamur.uclouvain.be; 5 Division of Pulmonology, CHU UCL Namur (Godinne Site), Université Catholique de Louvain (UCLouvain), Avenue G. Thérasse, 1, 5530 Yvoir, Belgium; lionel.pirard@chuuclnamur.uclouvain.be (L.P.); caroline.dahlqvist@chuuclnamur.uclouvain.be (C.D.); fabrice.duplaquet@chuuclnamur.uclouvain.be (F.D.); sebahat.ocak@chuuclnamur.uclouvain.be (S.O.); 6 Division of Nuclear Medicine, CHU UCL Namur (Godinne Site), Université Catholique de Louvain (UCLouvain), Avenue G. Thérasse, 1, 5530 Yvoir, Belgium; thierry.vanderborght@chuuclnamur.uclouvain.be; 7 Division of Pulmonology, Cliniques Universitaires Saint-Luc, Université Catholique de Louvain (UCLouvain), Avenue Hippocrate, 10, 1200 Bruxelles, Belgium; antoine.froidure@uclouvain.be; 8 Pole of Pulmonology, Institut de Recherche Expérimentale et Clinique (IREC), Université Catholique de Louvain (UCLouvain), Avenue Hippocrate, 55/B1.55.02, 1200 Bruxelles, Belgium; 9 Division of Thoracic Surgery, CHU UCL Namur (Godinne Site), Université Catholique de Louvain (UCLouvain), Avenue G. Thérasse, 1, 5530 Yvoir, Belgium; benoit.rondelet@chuuclnamur.uclouvain.be; 10 Division of Radiation Therapy, CHU UCL Namur (Sainte-Elisabeth Site), Université Catholique de Louvain (UCLouvain), Place Louise Godin, 15, 5000 Namur, Belgium; jean.vanderick@chuuclnamur.uclouvain.be (J.V.); vincent.remouchamps@chuuclnamur.uclouvain.be (V.R.)

**Keywords:** lung cancer, interstitial lung disease, retrospective, treatment, outcome

## Abstract

**Simple Summary:**

Interstitial lung disease (ILD) is associated with a higher risk of lung cancer. The impact of underlying ILD on cancer’s clinical characteristics, treatment strategies, and outcome is unclear, particularly in Caucasian populations. In this article, we reviewed the files of all patients diagnosed with lung cancer in a 38-month period and compared the patients with and without underlying interstitial changes for population and lung cancer characteristics, treatment and clinical outcome. Seven percent of patients with lung cancer had associated ILD at diagnosis. They were significantly older, but no other significant difference in population or cancer characteristics was observed. Patients with combined lung cancer and ILD had a worse clinical outcome even when similar treatment modalities were offered. Therefore, the choice of aggressive treatment strategies needs to be made carefully, with an awareness of the risk of acute exacerbation of ILD.

**Abstract:**

Background: Interstitial lung disease (ILD) is associated with a higher lung cancer (LC) risk and may impact cancer’s clinical characteristics, treatment strategies, and outcomes. This impact’s extent is unclear, particularly in Caucasians. Methods: In this retrospective observational study, we reviewed the files of all LC patients diagnosed in a 38-month period. Expert radiologists reviewed the computed tomography scans performed at diagnosis. Patients with LC and ILD (*n* = 29, 7%) were compared to those without ILD (*n* = 363, 93%) for population and cancer characteristics, treatments, and clinical outcomes. Results: Patients with LC and ILD were older (73 ± 8 vs. 65 ± 11 years; *p* < 0.001). There was no significant difference in LC histology, localization, stage, or treatment modalities. The respiratory complication rate after cancer treatment was significantly higher in the ILD group (39% vs. 6%; *p* < 0.01). Overall survival rates were similar at 12 (52% vs. 59%; *p* = 0.48) and 24 months (41% vs. 45%; *p* = 0.64) but poorer in the ILD group at 36 months, although not statistically significant (9% vs. 39%; *p* = 0.06). The ILD group had a higher probability of death (hazard ratio (HR) = 1.49 [0.96;2.27]), but this was not statistically significant (*p* = 0.06). In a Cox regression model, patients with ILD treated surgically had a significantly higher mortality risk (HR = 2.37 [1.1;5.09]; *p* = 0.03). Conclusions: Patients with combined LC and ILD have worse clinical outcomes even when similar treatment modalities are offered.

## 1. Introduction

Interstitial lung disease (ILD) is defined as a process of pulmonary inflammation and scarring that can result into lung fibrosis. It can be caused by various etiologies such as infection, drugs, radiation, systemic diseases, or toxic exposition, but ~60% are idiopathic as in idiopathic pulmonary fibrosis (IPF). The estimated incidence of ILD is 30 [1] and that of IPF is 0.22 to 7.4 per 100,000 cases per year [2]. Prognosis varies according to etiology, the worst being for IPF with a median overall survival (OS) of 3 years [3]. Patients with ILD have an increased risk of developing lung cancer, the prevalence being 5% to 48% [4]. The risk is particularly higher for IPF, with a relative risk of 4.96 [5] and 17% of patients dying of lung cancer [4]. The association of lung cancer and ILD may have an impact on cancer’s clinical characteristics, treatment strategies, and outcomes but the extent of this impact is still unclear, particularly in Caucasian populations. Previous studies on the topic frequently focused on IPF or on a specific cancer treatment modality. Moreover, they often included a limited number of patients, lacked a control group, and were conducted on Asian populations. In the present study, we aimed to evaluate the impact of ILD on the clinical presentations, treatments, and outcomes of Caucasian patients with lung cancer.

## 2. Materials and Methods

### 2.1. Study Population

We retrospectively reviewed the files of all patients diagnosed with lung cancer between January 2016 and March 2019 at CHU UCL Namur (Godinne Site), a Belgian university hospital and lung transplantation center. All chest computed tomography (CT) scans performed at the time of diagnosis were reviewed by two radiologists (K.R. with 5 years of chest CT experience, and M.V.D., a thoracic radiologist, with 15 years of chest CT experience) for signs of ILD (reticulations, ground-glass opacities, lung nodules, and honeycombing). When such signs were present, a specific diagnosis of ILD was made based on histology or radiological aspects and clinical context. Patients with lung cancer combined with ILD (*n* = 29, 7%) were compared to those with lung cancer but no ILD (*n* = 363, 93%) for population characteristics, lung cancer characteristics, lung cancer treatment, and clinical evolution.

### 2.2. Clinical Data

Clinical and survival data for all patients were extracted from the tumor registry and hospital charts. Tumor localization and staging were based on CT or positron emission tomography (PET)–CT imaging at diagnosis. The histological classification of tumors was based on the World Health Organization criteria [6]. Lung cancer staging was based on the eighth TNM staging system of the International Association for the Study of Lung Cancer [7]. Non-squamous non-small-cell lung cancers (NSCLC) were tested for genetic mutations via DNA next-generation sequencing (NGS) and for common genetic translocations via immunohistochemistry followed by fluorescent in situ hybridization when necessary or via RNA NGS. Spirometry and the diffusion capacity of the lung for carbon monoxide (DLCO) were measured according to the European Respiratory Society (ERS) recommendations and the results are expressed as the percentage of normal predicted values. The prescribed treatment was considered t standard-of-care (SOC) when it matched the gold-standard treatment, as defined by the European Society for Medical Oncology (ESMO), based on tumor histology and staging. OS was defined as the time between lung cancer diagnosis and death. This study was approved by the institutional ethical review board.

### 2.3. Statistical Analysis

Statistical analyses were performed using 4.1.0 R software (Computing Software for Statistics, Vienna, Austria) and the Tableone package. Continuous variables were presented as mean ± standard deviation (SD) or as median and interquartile range [IQR], and compared using the unpaired *t*-test. Categorical variables were expressed as percentages and compared with the chi-square test or Fisher’s exact test. Survival between fibrotic and control patients was described using Kaplan–Meier survival analysis and was assessed via Cox models. All *p* values were two-tailed with statistical significance set at *p* < 0.05; 95% confidence intervals are shown.

## 3. Results

### 3.1. Patient Characteristics

In total, 29 patients were included in the lung cancer and ILD group and 363 were included in the lung cancer and non-ILD group. The baseline characteristics of both patient groups are summarized in Table 1. Patients with lung cancer and ILD were significantly older at diagnosis (73 ± 8 vs. 65 ± 11 years; *p* < 0.01). There were more males in the ILD group but this was not statistically significant (72% vs. 63%; *p* = NS). The cumulative smoking history was similar in both groups (40 (25,50) vs. 40 pack years (30,50), *p* = 0.64), but more patients were active smokers at diagnosis in the non-ILD group (21% vs. 47%; *p* < 0.05). Spirometry values were similar in both groups, while patients with underlying ILD had a significantly lower DLCO (59% (45,67) vs. 74% (59,88); *p* < 0.01).

Associated parenchymal changes observed in the systematic review of chest CT-scans and diagnoses of associated ILD are represented in Table 2. Among patients presenting signs of ILD on a chest CT scan, about half had a diagnosis based on histology (*n* = 13/29; 45%) and about half (*n* = 12/29; 41%) presented IPF (*n* = 9/29; 31%) or combined pulmonary fibrosis and emphysema (CPFE) (*n* = 3/29; 10%). Over one-third of patients (*n* = 11/29; 38%) had honeycomb changes on chest CT scans. Exposition-related ILD included cases of medication-induced (amiodarone) ILD, radiation-induced lung injury, and pneumoconiosis. Systemic disease-related ILD included interstitial lung changes associated with rheumatoid arthritis, systemic sclerosis, and sarcoidosis. The ILD was of a fibrosing pattern in 66% of the patients (*n* = 19/29). Notably, the presence of interstitial changes was acknowledged in 62% (*n* = 18/29) of patients’ files. Only one patient (*n* = 1/29; 3%) was treated with anti-fibrotic drugs.

### 3.2. Lung Cancer Characteristics

Cancer characteristics in both patient groups are summarized in Table 3. There was no significant difference in lung cancer histological subtypes or localization. Only one patient with non-squamous NSCLC in the ILD group (*n* = 1/14; 7%) had a targetable genetic mutation (KRAS G12C), presenting a lower prevalence than that in the non-ILD group (*n* = 41/214; 19%) but this was not statistically significant (*p* = 0.26). We noticed a greater proportion of locally advanced stages (III) at diagnosis in the ILD group and more metastatic stages (IV) in the non-ILD group, but the difference was not statistically significant either. The proportions of early stage cancers were similar in both groups.

### 3.3. Cancer Treatments and Respiratory Complications

Lung cancer treatment modalities are summarized in Table 4. Similar proportions of patients were offered first-, second-, or even third-line treatment and a comparable fraction of patients received SOC therapy. There was no significant difference between the two groups in terms of treatment modalities (surgery (conservative or not), radiation therapy (stereotactic radiotherapy or conventional thoracic radiotherapy combined with chemotherapy), or systemic therapy). However, there was a significant difference in the respiratory complication rate after cancer treatment, which was higher in the ILD group (39% vs. 6%; *p* < 0.01). Respiratory complications included infections (*n* = 1 in the ILD group vs. *n* = 0 in the non-ILD group), radiation-induced lung injury (*n* = 2 vs. *n* = 7), immune-related pneumonitis (*n* = 1 vs. *n* = 6), acute respiratory distress syndrome (*n* = 0 vs. *n* = 5), or medication-related pneumonitis (*n* = 0 vs. *n* = 2). Sixty percent of respiratory complications in patients with ILD were labeled as an acute exacerbation (AE) of lung fibrosis (*n* = 6). They were triggered by radiotherapy (*n* = 3) as well as surgery (*n* = 2) or immune checkpoint inhibitor (ICI) therapy (*n* = 1). Due to the low patient numbers, it was not possible to determine if one treatment modality induced more AEs than the others did.

### 3.4. Survival

**Figure 1:**
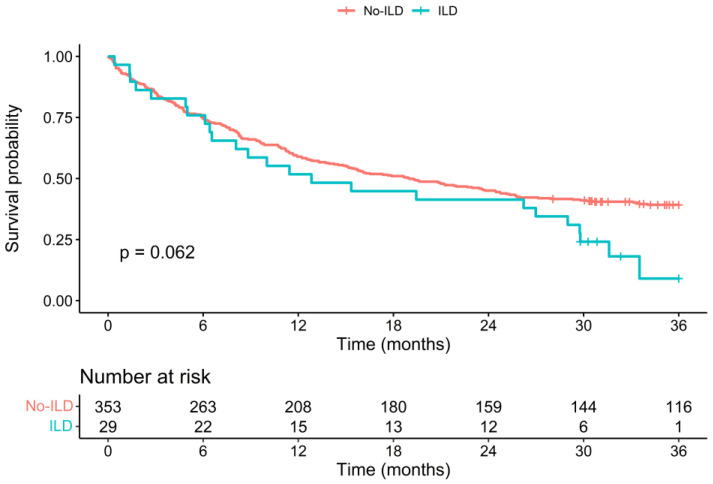
Kaplan–Meier survival curves of survival data of patient groups with ILD and without ILD, with a follow-up duration of 36 months after cancer diagnosis. The *p*-value is from a logrank test. Abbreviations: ILD: interstitial lung disease; *p*: *p* value.

**Figure 2:**
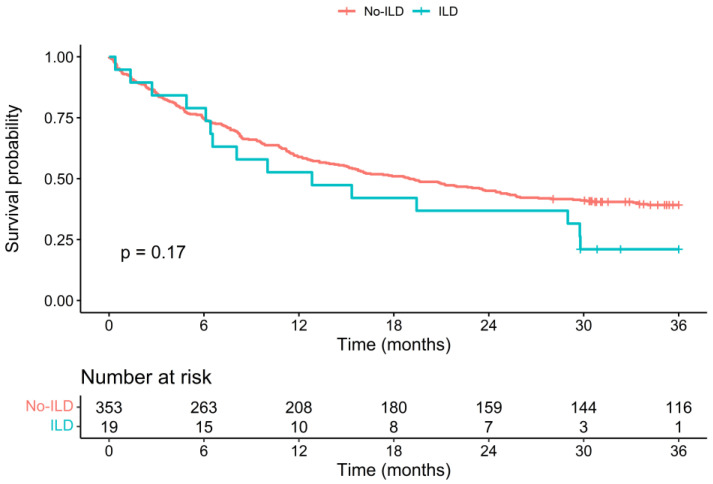
Kaplan–Meier survival curves of survival data of patient groups with ILD of a fibrosing pattern and without ILD, with a follow-up duration of 36 months after cancer diagnosis. *p* value is from a logrank test. Abbreviations: ILD: interstitial lung disease; *p*: *p* value.

The Kaplan–Meier survival curves of both patient groups are shown in Figure 1. While the survival curves follow a similar course initially, they markedly separate after 24 months to the detriment of the ILD group. Patients in the ILD group had a higher probability of death (hazard ratio (HR) = 1.49 [0.96;2.27]), although this was not statistically significant *p* = 0.06). The 12-month OS rate was 52% in the ILD group and 59% in the non-ILD group (*p* = 0.48), while the 36-month OS rate was 9% in the ILD group and 39% in the non-ILD group (*p* = 0.062). The median OS was 12.8 months in the ILD group [8.0–29.8] and 19 months in the non-ILD group [15.1–24.5]. The Kaplan–Meier survival curves and OS rates are similar for patients with an ILD of a fibrosing pattern (Figure 2).

**Figure 3:**
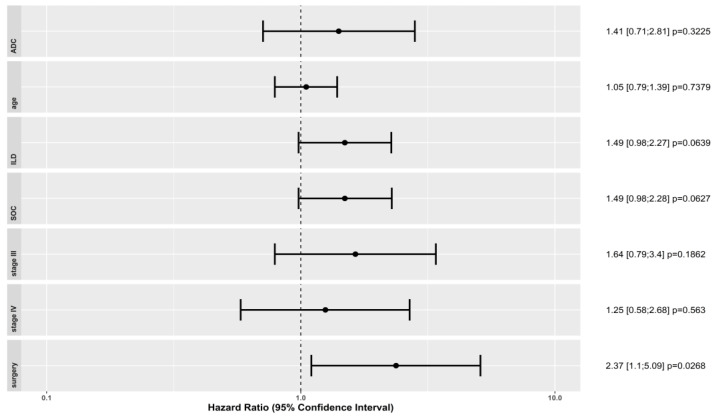
The Cox proportional-hazards model represents hazard ratio of mortality between the non-ILD and ILD groups. We represent the hazard ratio and 95% confidence intervals associated with age, adenocarcinoma histology, tumor stage III, tumor stage IV, surgical treatment, and standard-of-care treatment. Abbreviations: ILD: interstitial lung disease; *p*: *p* value; SOC: standard-of-care.

We used a Cox regression model to determine if some patient (age) or cancer characteristics (adenocarcinoma histological subtype, and stage III or IV cancer) or treatments (surgery, or SOC) had an impact on the OS difference observed between both groups. Results are shown in Figure 3. Patients with lung adenocarcinoma or with lung stage III or IV cancer did not present a significant OS difference between the ILD and non-ILD groups. However, patients with ILD who were treated surgically had a significantly higher risk of mortality than those in the non-ILD group did (HR = 2.37; *p* = 0.03). Other histologies, lung cancer stages, or treatment modalities could not be evaluated in this regression model due to the low number of patients. The impact of radiation therapy, in particular, could not be evaluated as it only concerned three patients of the ILD group. The risk of mortality was higher in patients who received SOC treatment if they had ILD, regardless of the treatment modality, although this was not statistically significant (HR = 1.49; *p* = 0.06). Age at diagnosis was not a determinant in terms of the risk of mortality (HR = 1.05; *p* = 0.73).

## 4. Discussion

Due to common risk factors and similarities in their physiopathological mechanisms, lung cancer is more frequent among patients with ILD [4]. Equally, higher proportions of patients with ILD are observed among lung cancer patients. In our study, 7% of all patients diagnosed with lung cancer presented radiologic signs of ILD, which is concordant with previously published data [8,9]. About half of our patients with lung cancer and ILD had IPF or CPFE, the two forms of ILD also most commonly associated with lung cancer in the literature [4]. It is important to highlight that the association of lung cancer and ILD was underdiagnosed, as associated ILD was mentioned in the medical files of only 62% of our patients with lung cancer and ILD. In a French retrospective study, associated ILD was mentioned in only 38% of patients’ files with lung cancer and ILD [8]. This under-diagnosis is problematic as associated ILD has a negative impact on treatment complication rates and lung cancer prognosis.

Lung cancer patients with associated ILD were older and the proportion of males tended to be more important, although the difference was not significant. This is concordant with the patient characteristics of the general ILD population. About one-third of patients with ILD had honeycomb changes on chest CT scans, which reflects fixed fibrotic changes and two-thirds presented with ILD with a fibrosing pattern. Patients with ILD had lower DLCO values on lung function tests but globally the lung function values were only mildly altered in both patient groups. This suggests a mild degree of severity of the underlying ILD, combined or not to other pulmonary pathologies. Concordantly, only one patient was treated with an anti-fibrotic drug. We suppose that the under-diagnosis of associated ILD, the very strict criteria for anti-fibrotic drug reimbursement and, probably, a limited extent of the fibrotic changes on CT scans explain why one IPF patient was treated with an anti-fibrotic drug.

Previous data in the literature described lung cancer in ILD patients as more frequently located in the lower pulmonary lobes, at the junction between the healthy lung and fibrosis, and as being of a squamous histological subtype [4]. In our population, we did not find significant differences in terms of tumor localization or cancer histology. Regarding the molecular profile of lung cancer, only one of our ILD patients with non-squamous NSCLC (7%) had a targetable genetic mutation (KRAS G12C), which tended to be lower than that in the non-ILD group (21.5%), without being significant (*p* = 0.26). Similarly, previous studies reported that patients with combined NSCLC and ILD have lower targetable mutation rates, especially for EGFR mutations [3].

Surprisingly, despite the presence of associated ILD, all our patients were offered comparable treatment strategies. There was no difference in the percentage of patients who were offered first-, second-, or third-line treatment or in the proportion of patients who received SOC treatment. Similarly, we did not observe higher rates of conservative surgery or stereotaxic radiation therapy in the ILD group. The absence of treatment adjustment in our patients with ILD may be surprising as it has previously been reported that patients with ILD are at a higher risk of complications, especially of an AE of lung fibrosis following all cancer treatment modalities [8]. This may be explained by the fact that the interstitial changes were relatively discreet in the majority of our patients, as only 38% of the ILD patients had honeycombing and interstitial changes were not reported in 38% of these patients. This highlights the necessity to acknowledge associated ILD at the time of cancer diagnosis to include it in therapeutic decision making, especially as, concordantly with the literature, we observed a higher rate of pulmonary complications following cancer treatment in patients with ILD (39% vs. 6%; *p* < 0.01). Pulmonary complications were observed with all the treatment modalities, except chemotherapy, but the low number of patients did not allow us to explore if one treatment modality came with a greater risk than the others did.

In a retrospective study evaluating the risk of AE after lung cancer surgery in patients with ILD, an AE rate of 9.3% and a secondary mortality rate of 44% were reported. The risk of AE was associated with ILD severity and the resected lung volume (wedge resection vs. segmentectomy or lobectomy (OR 3.83 (1.94–7.57)) [10]. Another Japanese retrospective study reported that thoracic radiotherapy in patients with comorbid ILD induced AE or radiation pneumonitis in 20–30% of patients, and that the risk was associated with the irradiated lung volume and the mean lung dose of radiation. The mortality risk during thoracic radiotherapy was 166 times higher in the case of associated ILD [9]. Similarly, a higher risk of radiation pneumonitis was reported after stereotactic body radiation therapy (SBRT) in patients with underlying ILD; in a retrospective study which identified signs of ILD in 20/159 patients who received SBRT for stage I NSCLC, multivariate analysis showed that ILD was a risk factor for radiation pneumonitis of ≥ grade 2 (HR 5.77 (2.72–12.3)) and that three-year OS was worse in the ILD group (53.8% vs. 70.8%; *p* = 0.28) [11]. The risk of AE due to chemotherapy in patients with associated ILD has been reported to be 10–30% [12]. Some chemotherapy agents, such as Gemcitabine, Amrubicin, and Irinotecan, have been identified as coming with a particularly high risk of AE. Patients with UIP are more at risk of AE than are non-UIP ILD patients (30% vs. 8%; *p* = 0.005) [12]. There are only limited data available concerning the use of immune checkpoint inhibitors (ICI) in patients with ILD, but the risk of immune-related pneumonitis seems to be higher [13]. Nevertheless, a multicentric retrospective study conducted in France showed that therapeutic response to nivolumab in advanced-stage NSCLC patients with underlying ILD was similar to that of the non-ILD population and concluded that ICI could be beneficial for these patients [14]. Our study reported two cases of respiratory complications labeled as immune-related pneumonitis or acute exacerbation in five patients in total with ILD who were offered treatment with an ICI. The limited number of patients treated with ICIs in our study may be explained by the fact that it is based on patients diagnosed with lung cancer between January 2016 and March 2019, at the beginning of the use of immunotherapy.

Concordantly with previously published data, we observed a tendency of poorer prognosis in patients diagnosed with lung cancer and underlying ILD. Surprisingly, the Kaplan–Meier survival curves of both patient groups followed a similar course for 24 months after diagnosis, and the curves split afterwards with an excess of mortality in the ILD group. While OS rates were similar between both groups at 12 and 24 months, the 36-month OS rate was lower, although not statistically significantly, in the ILD group compared to that in the non-ILD group (9% vs. 39%; *p* = 0.06). Similarly, in a retrospective observational study comparing 49 patients with lung cancer and ILD to 145 matched control cases, the median OS was significantly shorter in ILD patients (*p* = 0.04) and the one- and two-year OS rates were significantly different (one-year OS: 44.7% vs. 63.1%, *p* = 0.03; and two-year OS: 19.2% vs. 37.9%, *p* = 0.01) (8). The differences between their results and ours may be due to differences in the proportions of IPF and other ILD subtypes among the ILD patients, as they impact the prognosis.

In another retrospective study comparing 122 patients with lung cancer and IPF to four control cases diagnosed with lung cancer, 82.8% of patients were offered a treatment [3]. As in our study, similar proportions of patients in both groups were offered surgery, radiation, or systemic therapy but, as opposed to our study, the percentage of conservative surgery and SBRT was more important in the IPF group (conservative surgery: 41.8% vs. 11.2%; *p* < 0.001. SBRT: 52.2% vs. 24.6%; *p* = 0.015). The five-year OS was significantly lower among patients with IPF (14.5% vs. 30.1%; *p* < 0.001) and the median survival time was significantly lower after surgical treatment (42 months vs. 90 months; *p* < 0.001) or radiotherapy (5 vs. 18 months; *p* < 0.001), which is concordant with our results, despite the fact that they underwent treatment adjustment while we did not.

Notwithstanding functionally mild ILD, patients with lung cancer and interstitial lung changes have a worse clinical outcome, even when similar treatment modalities are offered. Despite the performance of radical treatment with a curative intent, the OS is lower if interstitial lung changes are present on chest CT scans. This highlights the importance of researching signs of ILD at the time of diagnosis of lung cancer, especially as it has been reported that associated ILD is underdiagnosed. Given the worse prognosis of patients with interstitial lung changes, the choice of aggressive treatment strategies needs to be made carefully. The survival of patients with associated ILD is particularly compromised in case of an AE of interstitial disease. The risk of such AEs is associated with all cancer treatment modalities, but some factors influencing this risk have been identified. Hence, the necessity of acting conservatively in the case of surgery and radiotherapy should be evaluated and chemotherapy and ICI should be used with caution. Given the particularly bad prognosis of IPF, with a median OS of three years, the relevance of curative intent treatments, especially if these patients are at high risk of AE, could even be questioned.

Our study has several limitations. First, the retrospective design implies that some of our patients had missing data. These missing data mostly covered descriptive population data such as smoking status or lung function tests and concerned a limited number of patients. Second, the monocentric character of our study limited the number of patients included and particularly the number of patients presenting an associated ILD, which limited the possibility to identify significant differences from our reference population, to evaluate population subgroups and to extrapolate results to larger populations.

In addition to the limitations, importantly, our study has several strengths. First, data analyzing the association of lung cancer and ILD in a Caucasian population are very limited. The association of ILD and lung cancer is rare and thus difficult to study, but as a lung transplantation center, our hospital concentrates on patients with a wide variety of lung pathologies such as ILD, which might have enhanced the chance to collect such patients. Our numbers reinforce most of the observations made in previous reports on both Caucasian and non-Caucasian populations. As data on the subject are very limited, we consider that corroborating previous results with ours is of clinical interest. Second, two radiologists systematically reviewed all CT scans made at the time of diagnosis, which ensured a more accurate assessment of ILD patterns. Third, we included patients treated with ICIs, while data concerning the use of ICIs in patients with underlying ILD is still very limited. Five of our patients with ILD received ICI therapy in first- or second-line treatment and were included in our evaluation of pulmonary complications. Finally, the unique point of view of our article is to base our patients’ population on all new diagnoses of lung cancer and to evaluate the impact of all types of ILD on a Caucasian population in a real-life setting. We also investigated if the presence of interstitial lung changes had an impact on therapeutic decision making and if patients with interstitial changes were offered standard-of-care therapy or more conservative options.

## 5. Conclusions

In conclusion, patients with combined lung cancer and ILD have a worse clinical outcome even when similar treatment modalities are offered. Therefore, the management of lung cancer in patients with underlying ILD should be carefully discussed in a multidisciplinary team, that is aware of the risk of an AE of ILD. Treatment options should also be thoroughly discussed with patients, as well as the risk of AE and its potentially fatal consequences. Further and prospective studies are needed to guide therapeutic decision making in these patients.

## Figures and Tables

**Table 1:** Baseline patient characteristics.

	ILD	Non-ILD	*p*
Patients (N)	29	363	-
Age (mean ± SD)	73 ± 8	65 ± 11	<0.01
Sex (male, %)	72	63	0.44
Smoking history (median [IQR], PY)	40 (30–50)	40 (25–50)	0.64
Active smoking (%)	21	47	0.01
Lung function tests			
FEV1 (median [IQR])	85 (64–103)	78 (60–91)	0.10
FVC (median [IQR])	87 (76–106)	95 (80–106)	0.44
DLCO (median [IQR])	59 (45–67)	74 (59–88)	<0.01

Abbreviations: DLCO: diffusion lung capacity of carbon monoxide; FEV1: forced expired volume in one second; FVC: forced vital capacity; ILD: interstitial lung disease; IQR: interquartile range; *p*: *p* value; PY: pack years; SD: standard deviation.

**Table 2:** Parenchymal changes observed on chest computed tomography scanner and ILD diagnoses.

Interstitial Images	ILD	Non-ILD	*p*
Emphysema (%)	72	47	<0.01
Ground-glass opacities (%)	79	6	<0.01
Honeycombing (%)	38	0	<0.01
Lung nodules (%)	10	4	0.12
Reticulations (%)	90	1	<0.01
**Diagnosis**			
CPFE (N, %)	4 (14%)		
Exposition-related (N, %)	4 (14%)		
Minimal fibrosis (N, %)	2 (7%)		
Systemic disease-related (N, %)	3 (10%)		
IPF (N, %)	9 (31%)		
Undetermined (N, %)	4 (14%)		
Other diagnosis (N, %)	4 (14%)		

Abbreviations—CPFE: combined pulmonary fibrosis and emphysema; ILD: interstitial lung disease; *p*: *p* value; IPF: idiopathic pulmonary fibrosis.

**Table 3:** Baseline cancer characteristics.

	ILD	Non-ILD	*p*
**Histology (N, %)**			0.57
Adenocarcinoma	12 (41%)	172 (47%)	
Squamous-cell carcinoma	9 (31%)	102 (28%)	
Small-cell lung cancer	6 (21%)	47 (13%)	
Other	2 (7%)	42 (12%)	
**Tumor localization (N, %)**			0.345
Upper/middle lobe	16 (55%)	224 (63%)	
Inferior lobe	13 (45%)	133 (37%)	
Left lung	15 (52%)	164 (46%)	0.664
**Tumor stage (N, %)**			0.067
0	0	7 (2%)	
I	8 (28%)	103 (28%)	
II	2 (7%)	26 (7%)	
III	11 (38%)	62 (17%)	
IV	8 (28%)	165 (46%)	

Abbreviations: ILD: interstitial lung disease; *p*: *p* value.

**Table 4:** Lung cancer treatment modalities.

	ILD	Non-ILD	*p*
**Cancer treatment**			0.49
Surgery (N, %)	12 (55%)	133 (42%)	
Pneumonectomy/lobectomy	9 (75%)	105 (80%)	0.96
Segmentectomy/wedge resection	3 (25%)	28 (20%)	
Radiotherapy (N, %)	3 (14%)	45 (14%)	
Radiochemotherapy	1 (33%)	25 (56%)	
SBRT	2 (67%)	13 (29%)	
Radiotherapy	0 (0%)	7 (16%)	
Systemic treatment (N, %)	7 (32%)	138 (44%)	
Chemotherapy	4 (57%)	99 (72%)	
Targeted therapy	0 (0%)	4 (3%)	
ICI therapy	1 (14%)	7 (5%)	
Chemotherapy + ICI therapy	2 (29%)	14 (10%)	
**Lines of treatment (N, %)**			
First-line	23 (79%)	317 (87%)	0.22
Second-line	10 (34%)	110 (30%)	0.64
Third-line	2 (7%)	44 (12%)	0.40
Standard-of-care treatment (N, %)	17 (59%)	241 (66%)	0.82
Early interruption of first-line treatment (N, %)	2 (9%)	25 (8%)	0.89
Pulmonary complications (N, %)	9 (39%)	18 (6%)	<0.01

Abbreviations—ICI: immune checkpoint inhibitor; ILD: interstitial lung disease; *p*: *p* value; SBRT: stereotactic body radiation therapy.

## Data Availability

Data are available on request due to privacy restrictions. The data presented in this study are available on request from the corresponding author. The data are not publicly available due to patient’s data confidentiality.
